# Single-cell transcriptomic data reveal the cellular heterogeneity of glutamine metabolism in gastric premalignant lesions and early gastric cancer: Single-cell transcriptomic data reveal the cellular heterogeneity of glutamine metabolism

**DOI:** 10.3724/abbs.2025061

**Published:** 2025-04-23

**Authors:** Qingfeng Ni, Jiawei Yu, Yuanjie Niu, Zhenwei Han, Boyang Hu, Yang Wang, Jianwei Zhu

**Affiliations:** Department of Gastrointestinal Surgery Affiliated Hospital of Nantong University Nantong 226001 China

**Keywords:** glutamine metabolism, gastric cancer, chronic atrophic gastritis, single-cell transcriptomics, ERO1LB, cellular heterogeneity

## Abstract

Glutamine metabolism is a hallmark of cancer metabolism. This study aims to perform a comprehensive and systematic single-cell profile of glutamine metabolism in premalignant and malignant gastric lesions. We use single-cell transcriptomics data from chronic atrophic gastritis (CAG) and early gastric cancer (EGC) lesions and investigate glutamine metabolism features at the single-cell level. Experiments are implemented to validate the expression and biological role of ERO1LB in gastric cancer (GC). A single-cell atlas based on 22511 cells from premalignant and early-malignant gastric lesions is established. Among these cells, epithelial cells constitute the dominant cell population in both CAG and EGC lesions. The activity of glutamine metabolism is higher in epithelial cells from EGC lesions than in those from CAG lesions. Among the epithelial cell subpopulations, glutamine metabolism is more active in the epithelial cell subpopulation cluster_4 in EGCs than in CAG lesions. As a key marker gene of this subpopulation, *ERO1LB* is experimentally proven to be overexpressed in human GC tissue lesions. In both *in vitro* and *in vivo* experiments, overexpression of ERO1LB in GC cells increases glutamine metabolism, facilitates cell growth and migration and prevents cell apoptosis, and vice versa. This study provides insight into the cellular heterogeneityof glutamine metabolism within the gastric mucosa in premalignant and malignant gastric lesions and identifies ERO1LB as a key orchestrator of glutamine metabolism, which may help to identify markers for GC prevention and contribute to our understanding of GC pathogenesis.

## Introduction

Chronic atrophic gastritis (CAG) is a gastric precancerous condition with an independent risk of developing into gastric cancer (GC) and constitutes the background for dysplasia and adenocarcinoma together with intestinal metaplasia and dysplasia [ 1, 2] . This disease presents chronic inflammation of the gastric mucosa, accompanied by the loss of gastric glandular cells and replacement by intestinal-type epithelium, pyloric-type glands, or fibrous tissue [3]. Among patients with CAG, the annual incidence (person-year) of GC is 0.1%–0.5% [4], and the cumulative 5-year incidence is 0.7%–10% [5]. Cell type changes play an essential role in the cascade from precancerous to malignant lesions. Nonetheless, the full spectrum of diverse cell types and their molecular features remain to be characterized in gastric premalignant and malignant lesions, hampering investigations of their roles in GC pathogenesis.

Glutamine metabolism is a hallmark of cancer metabolism [ 6, 7] . Proliferating cancer cells depend largely upon glutamine for survival and proliferation [ 8, 9] . Glutamine is the most abundant circulating amino acid in the blood and muscle and is essential for distinct basic cellular functions of tumor cells, *e*. *g*., the synthesis of metabolites maintaining mitochondrial metabolism; antioxidant production for eliminating reactive oxygen species; the synthesis of non-essential amino acids, purines, pyrimidines and fatty acid components for cell replication; and the activation of cell signals [ 10– 13] . Glutamine metabolism has been shown to mediate CAG-induced carcinogenesis, but the molecular mechanisms governing this process remain to be explored [14]. Given the pleiotropic roles of glutamine in tumor cells, an in-depth understanding of glutamine metabolism is critical for the discovery of metabolism-based treatments for GC. Identification of the cells responsible for gastric tumorigenesis is important for understanding the molecular underpinnings of tumor evolution. Single-cell transcriptomics is a powerful tool for deciphering the cellular and molecular landscape at single-cell resolution, unlike bulk transcriptomics, which provides averaged data [15]. The application of single-cell transcriptomics has revolutionized our understanding of the biological features and dynamics within tumor lesions [ 16– 18] .

Hence, this study presents a single-cell map of glutamine metabolism in the gastric mucosa of patients with premalignant and malignant gastric lesions and reveals that ERO1LB is a key orchestrator that modulates glutamine metabolism.

## Materials and Methods

### Single-cell transcriptomics

Single-cell transcriptomics data on gastric antral mucosa biopsies from three CAG samples and one early gastric cancer (EGC) sample were acquired from the GSE134520 dataset ( https://www.ncbi.nlm.nih.gov/geo/query/acc.cgi?acc=GSE134520) [19].

### Quality control and preprocessing

Based upon DropletUtils package [20], the expression traits of ambiant RNA were assessed. The unique molecular identifier (UMI) threshold was set to 200, and UMI < 200 was defined as ambient RNA. The sum of the UMI number of the same gene in each droplet was the UMI number of the gene in the ambient RNA expression profile. The *P* value of each barcode was calculated via Monte Carlo. Barcodes with significant differences were regarded as real cells. Combined with the inflection point of the barcode rank curve, a barcode with a large total UMI was retained. The Scater toolkit was subsequently utilized for quality control [21]. The expression traits were determined according to the perCellQCMetrics function. Cells that expressed > 10% mitochondrial genes and < 10% ribosome genes were filtered out. Doublets were detected and removed via DoubletFinder [22]. The filtered data were combined and normalized via the Harmony function.

### Dimension reduction analysis

Single-cell data were linearly scaled with the ScaleData function from Seurat. Principal component analysis (PCA) was subsequently executed, which was corrected by running Harmony. Principal components with larger standard deviations were selected.

### Cell clustering and annotation

By running the FindNeighbors and FindClusters functions from the Seurat package, single cells were clustered, with subsequent dimension reduction via UMAP. With FindMarkers, marker genes of each cluster were screened under the following criteria: log2(fold change) > 0.1, cell cluster ratio < 0.01 and *P*  < 0.05. Cell type recognition was carried out for cell clustering via the SingleR package [23], and the cells were labelled in accordance with existing marker genes from the CellMarker database [24].

### Analysis of glutamine metabolism activity

The AUCell package was adopted for estimation of the activity of glutamine metabolism in each cell on the basis of the expression profiles of each cell and the gene set of glutamine metabolism [25].

### Functional enrichment analysis

Functional enrichment analysis of specified genes was conducted via the Gene Ontology and Kyoto Encyclopedia of Genes and Genomes (KEGG) pathway databases [ 26– 28] . By use of gene set variation analysis (GSVA) [29], hallmark pathway activity was estimated on the basis of expression profiling and the gene sets of hallmarks that were gathered from the Molecular Signatures Database [30].

### Analysis of transcription factor regulation and cell-cell interactions

The SCENIC computational approach was executed for simultaneously reconstructing gene regulatory networks and identifying the cell state [31]. Using the CellChat package [32], communication between cells was inferred on the basis of the expression profiles of receptor ligand genes corresponding to diverse cell types. Subsequently, cell-to-cell receptor and ligand pair networks were drawn.

### Patients and specimens

Human GC lesion and paracancerous normal tissue samples were obtained from the Affiliated Hospital of Nantong University. None of the patients received any treatment prior to tumor resection. All participants provided written informed consent. The experimental procedures complied with the Declaration of Helsinki. The project was approved by the Medical Ethics Committee of the Affiliated Hospital of Nantong University (2024-L103).

### Immunohistochemical staining

Following deparaffinization and rehydration, the formalin-fixed tissue sections were subjected to antigen retrieval. Endogenous peroxidase activity was quenched with 3% hydrogen peroxide, with subsequent blockade with 10% goat serum for 1 h at room temperature. The samples were incubated with a primary antibody against ERO1LB (1/100; ab197290; Abcam, Cambridge, USA) overnight at 4°C. Horseradish peroxidase-conjugated secondary antibody (1/500; ab7090; Abcam) was subsequently added, and the samples were incubated for 1 h at room temperature. Sections were developed via SignalStain DAB substrate (CST, Danvers, USA), which was then scanned and observed under an IX-73 microscope (Olympus, Tokyo, Japan).

### Cell culture

The normal gastric mucosa epithelial cell line GES-1 and two GC cell lines, SGC7901 and MGC803, were acquired from the Shanghai Institutes for Biological Sciences (Shanghai, China). All the cells were maintained in RPMI 1640 medium (Thermo Fisher Scientific, Waltham, USA) supplemented with 10% FBS, 100 U/mL penicillin and 100 U/mL streptomycin in a humidified incubator with 5% CO _2_ at 37°C.

### Western blot analysis

Proteins were extracted via radio-immunoprecipitation assay lysis reagent and quantified using a bicinchoninic acid kit (Thermo Fisher Scientific). After 8%–12% sodium dodecyl sulfate-polyacrylamide gel electrophoresis (SDS-PAGE), 30 μg of protein was electroblotted onto a polyvinylidene fluoride membrane (Millipore, Billerica, USA). Primary antibodies against ERO1LB (1/1000; ab197290; Abcam), glutaminase (GLS; 1/1000; ab156876; Abcam), glutamate dehydrogenase (GDH; 1/2000; ab170895; Abcam) or GAPDH (1/2500; ab9485; Abcam) were incubated with the membranes overnight at 4°C. Horseradish peroxidase-labelled secondary antibody (1/5000; ab7090; Abcam) was utilized for a 1-h of incubation. The protein bands were visualized via enhanced chemiluminescence reagent (Yeasen, Shanghai, China). The band intensity was quantified via ImageJ software (NIH, Bethesda, USA).

### Cell transfection

The designed and synthesized siRNAs against ERO1LB (si-ERO1LB) were acquired from Genechem Co., Ltd. (Shanghai, China). The sequence for si-ERO1LB-1 was 5′-GCCCUGAAGAUAUUAUUCUTTAGAAUAAUAUCUUCAGGGCTT-3′, the sequence for si-ERO1LB-2 was 5′-CUGGGCAAGAUAUGAUGAUTTAUCAUCAUAUCUUGCCCAGTT-3′, the sequence for si-ERO1LB-3 was 5′-CAAGCCUCGAUCUGUUUAUTTAUAAACAGAUCGAGGCUUGTT-3′, and the sequence for si-NC was 5′-UUCUCCGAACGUGUCACGUTTACGUGACACGUUCGGAGAATT-3′. The sequence of *ERO1LB* was amplified and cloned and inserted into a pcDNA3.1 vector to establish an ERO1LB overexpression (OE-ERO1LB) vector (Genechem Co., Ltd.). Lipofectamine 3000 (Thermo Fisher Scientific) was utilized for the transfection of siRNAs or plasmids according to the manufacturer’s instructions. For stably transfected cell lines, cells were transfected with lentiviruses expressing negative control or shRNA constructs targeting *ERO1LB*. The sequence for shERO1LB was 5′-GGAGCAATTAACAGCACATTA-3′, and the sequence for sh-NC was 5′-TTCTCCGAACGTGTCACGT-3′. shRNAs were transfected with polybrene (5 mg/mL; Sigma, St Louis, USA) at a multiplicity of infection (MOI) of 10. Stable cell lines were selected by treatment with puromycin (10 μg/mL) for 3 consecutive days after 72 h of transfection.

### Real-time reverse transcriptase-polymerase chain reaction (RT-qPCR)

Trizol reagent (Yeasen, Shanghai, China) was used for the extraction of total RNA. To detect mRNA, a reverse transcription kit was used to obtain cDNA. The SYBR ^®^ Premix Ex Taq ^TM^ II kit (TaKaRa, Dalian, China) was used for qPCR, which was conducted on an ABI 7500 qPCR instrument (ABI, Foster City, USA). Using *GAPDH* as an internal reference, the 2 ^−ΔΔCt^ method was utilized for the quantification of *ERO1LB* expression. The primer sequences for *ERO1LB* and *GAPDH* were as follows: *ERO1LB*, 5′-TTCTGGATGATTGCTTGTGTGAT-3′ (forward), 5′-GGTCGCTTCAGATTAACCTTGT-3′ (reverse); and *GAPDH*, 5′-GGAGCGAGATCCCTCCAAAAT-3′ (forward), 5′-GGCTGTTGTCATACTTCTCATGG-3′ (reverse).

### Transwell assay

Cell migration was measured via Transwell chamber assays without Matrigel. A total of 2 × 10 ^4^ cells were added to the upper chamber of the Transwell system (8 μm pore; BD Biosciences, Franklin Lakes, USA), and culture medium plus 20% FBS was added to the bottom chamber. After 24 h of culture, the migrated cells were fixed with 100% methanol and stained with 0.1% crystal violet (Sigma). Photographs were finally acquired with an IX-73 microscope (Olympus, Tokyo, Japan) and the numbers of migrated cells were counted.

### 5-Ethynyl-20-deoxyuridine (EdU) incorporation assay

An EdU incorporation assay was performed to detect the specific proliferation rates of GC cells with a Cell-Light EdU DNA Cell Proliferation kit (Beyotime, Shanghai, China) following the manufacturer’s instructions. The number of proliferating cells in three random fields was counted under a fluorescence microscope.

### Flow cytometry

Cell apoptosis was tested via an Annexin V-FITC apoptosis detection kit (eBioscience, San Diego, USA). The cells were collected and rinsed with PBS. The supernatants were removed via centrifugation. Next, 195 μL of binding buffer was used to re-suspend the samples, which were subsequently incubated with 5 μL of Annexin V-FITC as well as 10 μL of propidium iodide staining solution away from the light for 15 min. The cell suspension reached 500 μL after the addition of binding buffer. Apoptosis was ultimately measured with a flow cytometer (BD Biosciences).

### Mouse models

All animal experiments were approved by the Institutional Animal Care and Use Committee at Nantong University. For the xenotransplantation model, 5 × 10 ^6^ stably transfected GC cells were injected subcutaneously into the bilateral dorsal flanks of 6-week-old male BALB/C nude mice (Jiangsu Jicui Yaokang Biotechnology Co., Ltd., Lianyungang, China), which were randomly divided into different groups, with 6 mice in each group. The subcutaneous xenografts were harvested and recorded five weeks later. The tumor volumes were calculated as (length × width ^2^/2). For the lymphatic metastasis model, 2.5 × 10 ^6^ GC cells in a volume of 50 μL of PBS were injected into the footpads of the mice. The tumors of the popliteal lymph nodes (LNs) were enucleated 4 weeks after inoculation. The volume of each LN was calculated as (length × width ^2^/2).

### Statistical analysis

Data are presented as the mean ± standard deviation (SD) and were analyzed using either the independent *t* test or one-way ANOVA. Categorical data were analyzed using the chi-square test. Survival rates were assessed via the Kaplan-Meier method and log rank test. Statistical analysis was conducted via R Studio and GraphPad Prism. Statistical significance was set at *P*  < 0.05.

## Results

### Epithelial cells constitute a dominant cell population in both CAG and EGC lesions

**Figure 1:**
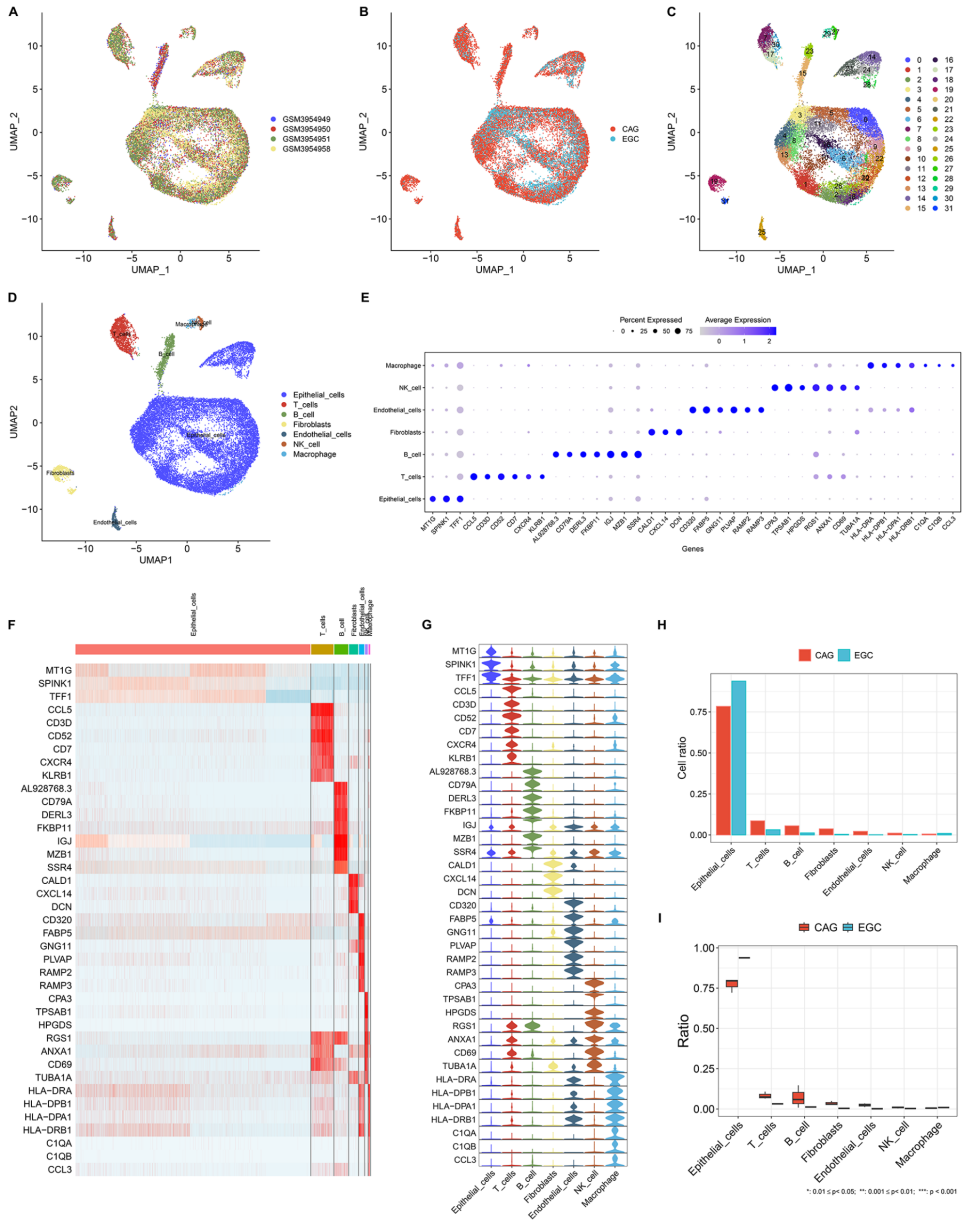
Figure 1 A single-cell transcriptome atlas of CAG and EGC patients (A,B) UMAP maps of three CAG and one EGC single-cell transcriptome samples. (C) Clustering analysis of 22511 filtered cells from premalignant and early-malignant lesions. (D) Recognition of cell types in accordance with the expression of existing marker genes. (E) Bubble chart visualizing the unique expression of the utilized marker genes in the recognized cell types. (F,G) Expression distribution of the marker genes across diverse cell types. (H,I) Comparison of the ratios of the cell types in CAG versus those in EGC.

We first analyzed single-cell transcriptome data from gastric antral mucosa biopsies from three CAG patients and one EGC patient. Following the removal of low-quality single cells ( Supplementary Figure S1A–E) and dimension reduction analyses ( Supplementary Figure S2A–E), UMAP profiles revealed that 22511 cells from premalignant and early-malignant gastric lesions were clustered into 32 cell clusters ( Figure 1A–C). Single-cell transcriptome atlas, which is composed primarily of epithelial cells ( *n* = 18213), T cells ( *n* = 1735), B cells ( *n* = 1089), fibroblasts ( *n* = 709), endothelial cells ( *n* = 414), NK cells ( *n* = 214) and macrophages ( *n* = 137) ( Figure 1D), was constructed on the basis of the expression of existing cell markers. The markers were almost uniquely expressed in the corresponding cell types: epithelial cells (MT1G, SPINK1 and TFF1), T cells (CCL5, CD3D, CD52, CD7, CXCR4 and KLRB1), B cells (AL928768.3, CD79A, DERL3, FKBP11, IGJ, MZB1 and SSR4), fibroblasts (CALD1, CXCL14 and DCN), endothelial cells (CD320, FABP5, GNG11, PLVAP, RAMP2 and RAMP3), NK cells (CPA3, TPSAB1, HPGDS, RGS1, ANXA1, CD69 and TUBA1A) and macrophages (HLA-DRA, HLA-DPB1, HLA-DPA1, HLA-DRB1, C1QA, C1QB and CCL3) ( Figure 1E–G). Notably, among the cell types, epithelial cells were the dominant cell type in both CAG and EGC lesions. The degree of cellular heterogeneity between premalignant and early-malignant lesions was also evaluated, and the ratio of epithelial cells was found to be strongly elevated in EGCs compared with that in CAGs ( Figure 1H,I). These findings indicate that epithelial cells in the microenvironment are possibly linked to gastric tumorigenesis.

### Glutamine metabolism is more active in epithelial cells in early-malignant lesions than in those in premalignant lesions

**Figure 2:**
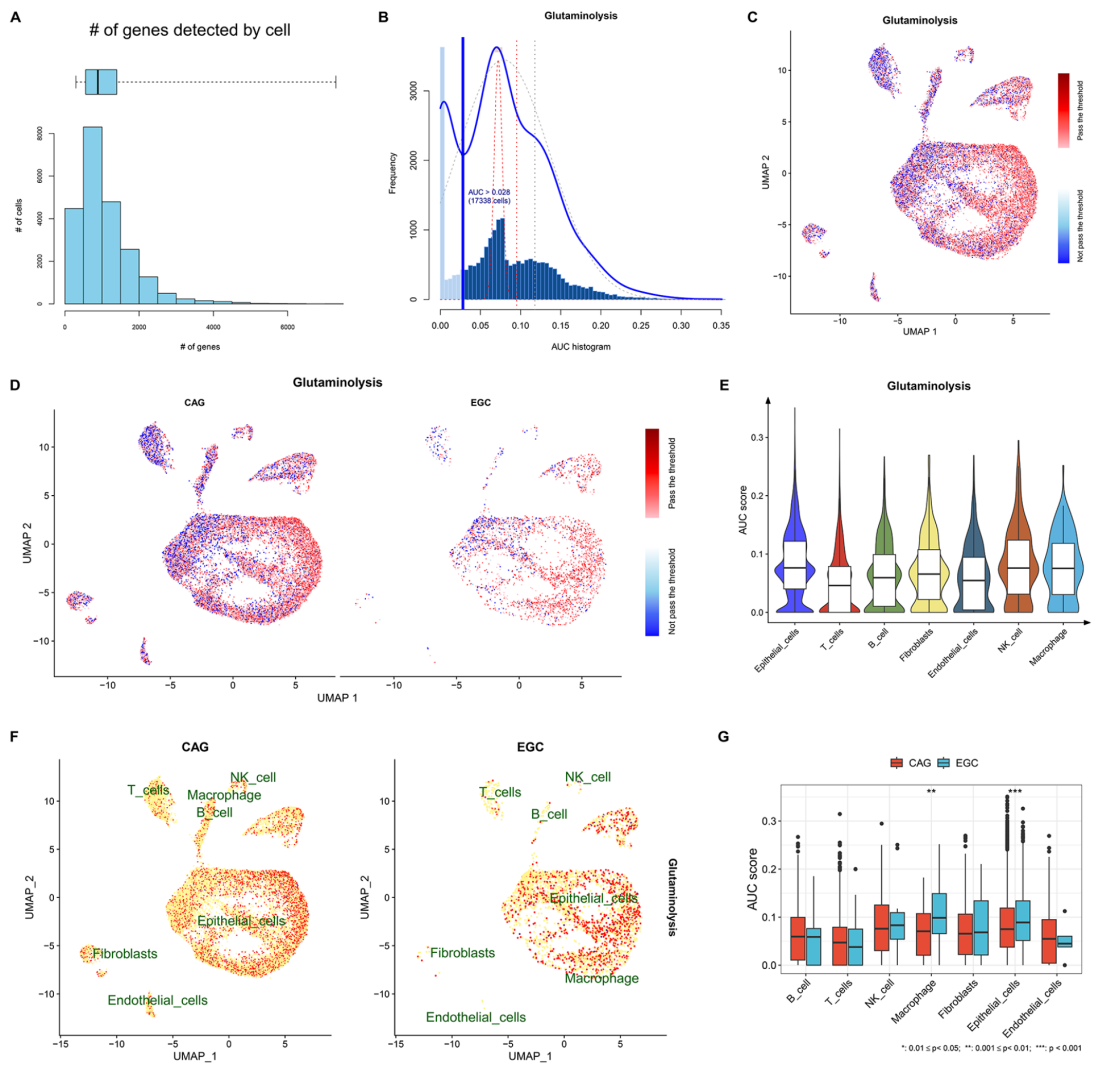
Figure 2 Heterogeneous glutamine metabolism across cell types in the inflammatory microenvironment between CAGs and EGCs (A) Number of cells that express glutamine metabolism genes. (B) Estimation of glutamine metabolism activity across cells. (C) UMAP map for the activity of glutamine metabolism across cells. (D) Glutamine metabolism activity in single cells from CAG and EGC. (E) Heterogeneous glutamine metabolism activity across diverse cell types. (F) Distribution of glutamine metabolism activity across CAG and EGC cell types. (G) Comparison of the activity of glutamine metabolism in each cell type between CAG and EGC. **P < 0.01, ***P < 0.001.

Glutamine metabolism is a hallmark of cancer metabolism [33]. The heterogeneity in glutamine metabolism was evaluated in both CAG and EGC lesions. On the basis of the expression of glutamine metabolism genes, glutamine metabolism activity in single cells was estimated ( Figure 2A,B). A notable distinction in glutamine metabolism activity was found between CAG and EGC lesions ( Figure 2C,D). Intriguingly, epithelial cells presented the highest activity of glutamine metabolism among all cell types ( Figure 2E). Moreover, heterogeneous glutamine metabolism activity in epithelial cells was found in CAG and EGC, with higher activity in EGC than in CAG ( Figure 2F,G). Thus, increased glutamine metabolism in epithelial cells might be associated with gastric tumorigenesis.

### Identification of heterogeneous epithelial cell subpopulations in both CAG and EGC lesions

**Figure 3:**
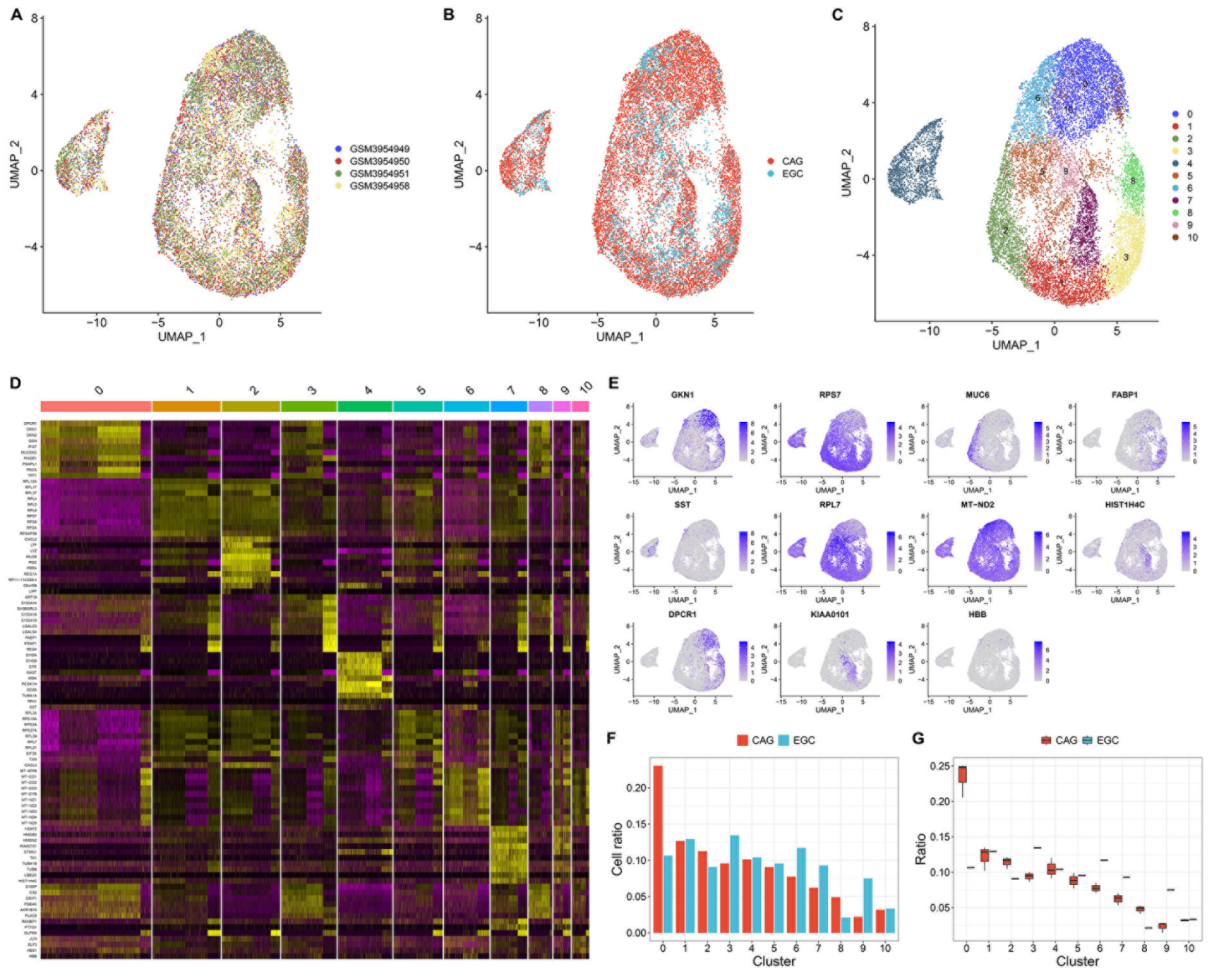
Figure 3 Epithelial cell subpopulations in CAG and EGC (A,B) UMAP plots of epithelial cells in three CAG lesions and one EGC lesion. (C) Clustering analysis of epithelial cell subpopulations. (D) Heatmap depicting the top ten marker genes in each epithelial cell subpopulation. (E) UMAP plots of the top marker genes in each epithelial cell subpopulation. (F,G) Comparison of the ratios of epithelial cell subpopulations between CAG and EGC lesions.

We focused on the heterogeneity of epithelial cells in the microenvironment of premalignant and early-malignant lesions ( Figure 3A,B). Eleven epithelial cell subpopulations were clustered ( Figure 3C). Subsequently, markers of each epithelial cell subpopulation were identified, *e*. *g*., GKN1, RPS7, MUC6, FABP1, SST, RPL7, MT-ND2, HIST1H4C, DPCR1, KIAA0101 and HBB, in clusters_0–10 ( Figure 3D,E). The heterogeneous epithelial cell subpopulations were monitored in both CAG and EGC lesions ( Figure 3F,G). Taken together, these findings indicate heterogeneity in epithelial cells in the microenvironments of CAG and EGC lesions.

### Glutamine metabolism is more active in the epithelial cell subpopulation cluster_4 in early-malignant lesions than in premalignant lesions

**Figure 4:**
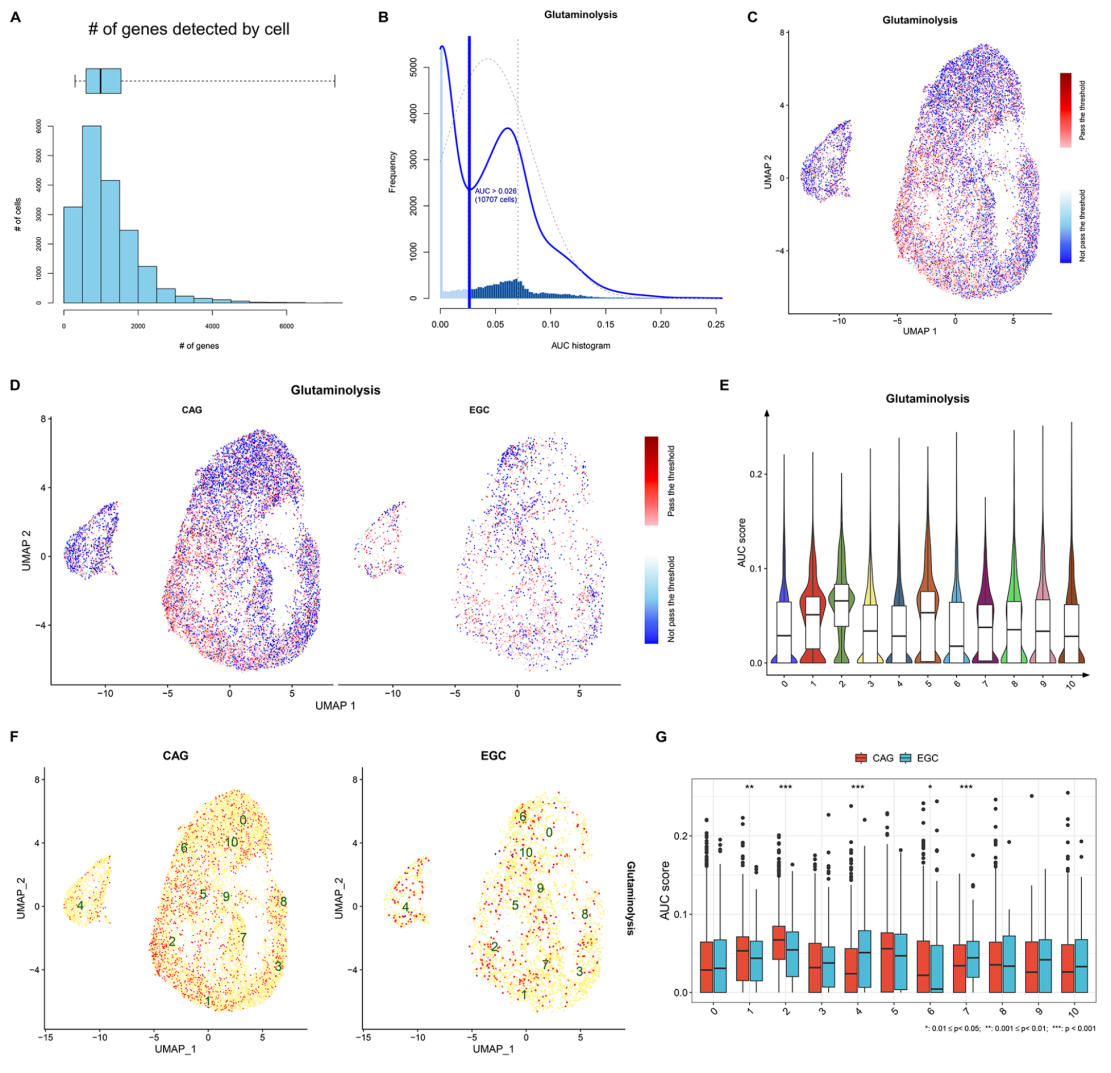
Figure 4 Heterogeneous glutamine metabolism in epithelial cell subpopulations between CAG and EGC (A) Number of epithelial cells that express glutamine metabolism genes. (B) Detection of glutamine metabolism activity across epithelial cells. (C,D) Distribution of glutamine metabolism activity across epithelial cells from CAG and EGC lesions. (E) Comparison of glutamine metabolism activity among diverse epithelial cell subpopulations. (F) Glutamine metabolism activity in different epithelial cell subpopulations from CAG and EGC lesions. (G) Comparison of glutamine metabolism activity in each epithelial cell subpopulation in CAG versus EGC. *P < 0.05, **P < 0.01, ***P < 0.001.

We further estimated the activity of glutamine metabolism in epithelial cells in both CAG and EGC lesions ( Figure 4A–D). Glutamine metabolism activity was found to be highly heterogeneous among the epithelial cell subpopulations ( Figure 4E). Compared with CAG, glutamine metabolism was more active in the epithelial cell subpopulation cluster_4 in EGCs ( Figure 4F,G). Hence, glutamine metabolism in the epithelial cell subpopulation cluster_4 might play a role in gastric tumorigenesis.

### Identification of markers and machinery of epithelial cell subpopulation cluster_4

**Figure 5:**
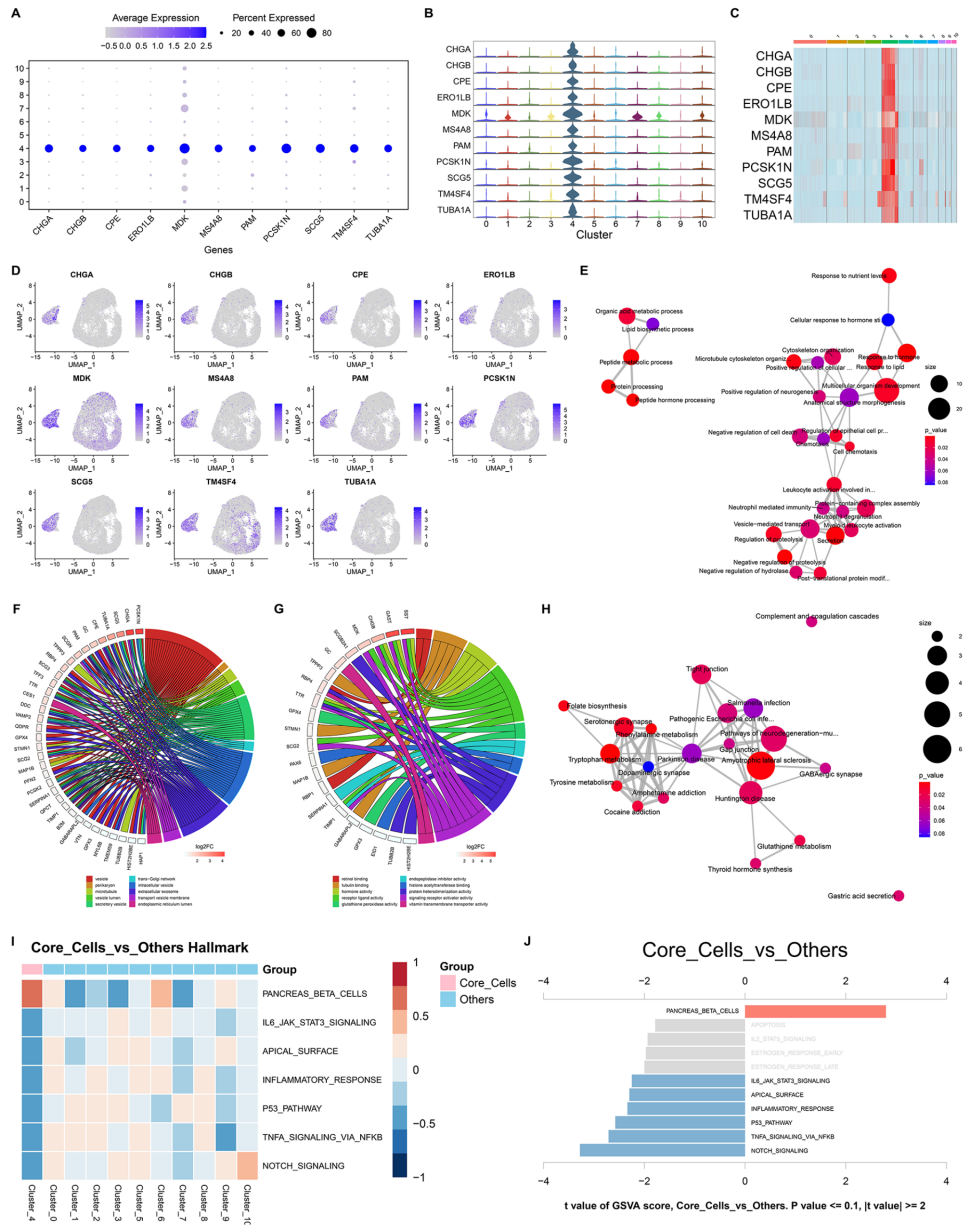
Figure 5 Identification of marker genes and machinery of epithelial cell subpopulation cluster_4 (A) Bubble diagram visualizing the unique expression of marker genes in epithelial cell subpopulation cluster_4. (B,C) Expression distribution of marker genes across epithelial cell subpopulations. (D) UMAP plots depicting the expression of genes in epithelial cells. (E) Biological process network based upon marker genes of cluster_4. (F,G) The main cellular components and molecular functions enriched by the marker genes. (H) KEGG pathway network based on the marker genes. (I) Distribution of the activity of hallmark pathways across epithelial cell subpopulations. (J) Comparison of the activity of hallmark pathways in cluster_4 versus other subpopulations.

CHGA, CHGB, CPE, ERO1LB, MDK, MS4A8, PAM, PCSK1N, SCG5, TM4SF4 and TUBA1A were uniquely expressed in the epithelial cell subpopulation cluster_4 and were regarded as key markers in this subpopulation ( Figure 5A–D). As illustrated in Figure 5E, these markers were strongly associated with protein processing and metabolism, cytoskeleton organization, cell death and immunity. In addition, these markers were strongly associated with vesicles, perikaryons, microtubules, extracellular exosomes, *etc*. ( Figure 5F), as well as retinol binding, tubulin binding, hormone activity, receptor ligand activity, *etc*. ( Figure 5G). Tryptophan, tyrosine and phenylalanine metabolism processes, *etc*., were notably enriched by the marker genes ( Figure 5H). These data revealed the key role of the marker genes in gastric tumorigenesis. Moreover, pancreas beta cells were uniquely active in cluster_4, whereas IL6-JAK-STAT3 signaling, the apical surface, the inflammatory response, the p53 pathway, and TNFA signaling via NF-κB and Notch signaling were uniquely inactive in this epithelial cell subpopulation ( Figure 5I,J). Collectively, the above findings clarified the marker genes and machinery unique to epithelial cell subpopulation cluster_4 in tumorigenesis.

### Transcriptional regulatory programs in epithelial cells from premalignant and early-malignant lesions

Next, distinctions in transcriptional regulatory programs in epithelial cells between premalignant and early-malignant lesions were assessed. Differentially expressed marker genes in epithelial cells were probed in two lesions. In total, 219 marker genes presented remarkable upregulation, whereas 249 marker genes presented notable downregulation in the epithelial cells of CAG versus those of EGC ( Supplementary Figure S3A). These marker genes were strongly connected to metabolic and immunity pathways ( Supplementary Figure S3B). Additionally, JUND (784 g), EGR1 (2426 g), MAZ (1753 g) and NPDC1 (191 g) presented notably lower regulon activity in epithelial cells of EGC than in those of CAG, with higher regulon activity of ETV4 (856 g), HDAC2 (495 g), HES6 (17 g), ONECUT2 (13 g), SOX4 (91 g), TAF7 (2850 g) and XBP1 (108 g) ( Supplementary Figure S3C–E). The heterogeneous expression of transcription factors was also found in distinct epithelial cell subpopulations ( Supplementary Figure S3F). Among the transcription factors, JUND and EGR1 were found to be universally expressed in all the subpopulations.

### Intercellular communication in the microenvironments of premalignant and early-malignant lesions

Among the ligand‒receptor pairs, epithelial cells, T cells, B cells, fibroblasts, endothelial cells, NK cells and macrophages closely interact in the microenvironments of premalignant and early-malignant lesions ( Supplementary Figure S4A,B). Tight communication between epithelial cell subpopulations was also detected ( Supplementary Figure S4C–F). These complex intercellular interactions might be involved in the pathogenesis of both CAG and EGC.

### ERO1LB is experimentally proven to be overexpressed in human GC lesions

**Figure 6:**
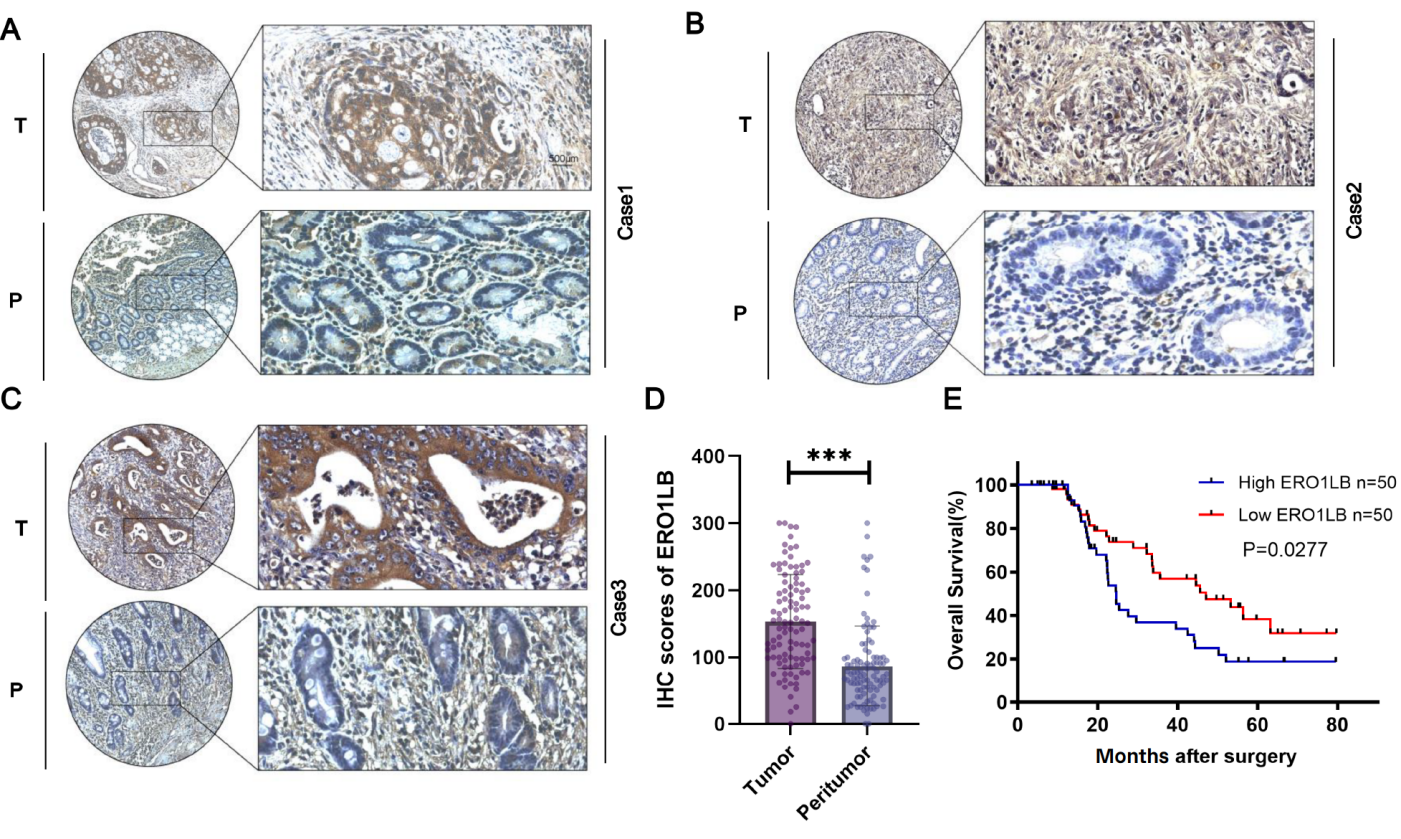
Figure 6 ERO1LB is notably overexpressed in human GC lesions (A–C) Immunohistochemical staining of ERO1LB expression in 3 pairs of human GC lesions and matched paracancerous tissues. (D) IHC score of 100 pairs of human GC tissues and matched paracancerous tissues. (E) Kaplan-Meier survival curves revealing that the survival rate of patients in the high ERO1LB expression group was significantly lower than that of patients in the low ERO1LB expression group. T, tumor tissue; P, paracancerous tissue ***P < 0.001.

ERO1LB (endoplasmic reticulum oxidoreductase 1 beta) enables thiol oxidase activity, facilitates insulin biogenesis and glucose homeostasis, and participates in mediating protein folding in the endoplasmic reticulum [ 34– 36] . Given the importance of the ERO1LB ^+^ epithelial cell subpopulation in GC, experimental verification was then carried out. We assessed the expression level of ERO1LB in 100 pairs of GC tissues and their corresponding adjacent tissues using immunohistochemical techniques and selected three pairs of typical representative stained images for presentation ( Figure 6A–D). We combined the subsequent analysis of the prognostic data of the patients and Kaplan-Meier survival curves, which revealed that the survival rate of patients in the high ERO1LB expression group was significantly lower than that of patients in the low ERO1LB expression group ( Figure 6E). Thus, elevated ERO1LB expression is linked to poor prognosis in patients with GC. The relationships between ERO1LB expression and clinicopathological features in GC patients are shown in Table 1. ERO1LB expression was strongly correlated with lymph node metastasis ( *P* = 0.004) and TNM stage ( *P* = 0.003). Nonetheless, there was no relationship with age, sex, tumor size, tumor site, or vascular invasion.

**Table:** **Table 1** Relationship between ERO1LB expression and clinico-pathological features in GC patients

Characteristics	ERO1LB		*P*
	Low expression ( *n* = 50)	High expression ( *n* = 50)	Total	
Age				
< 60	21	20	41	0.839
≥ 60	29	30	59
Gender				
Male	28	26	54	0.688
Female	22	24	46
Tumor size				
< 3cm	27	19	46	0.108
≥ 3cm	23	31	54
Tumor site				
Proximal	25	28	53	0.548
Non-proximal	25	> 22	47
Lymph node metastasis				
N0	27	13	40	0.004
N1–N3	23	37	60
TNM stage				
I–II	30	15	45	0.003
III	20	35	55
Vascular invasion				
Negative	26	19	45	0.159
Positive	24	31	55

### ERO1LB enhances glutamate metabolism in GC cells

**Figure 7:**
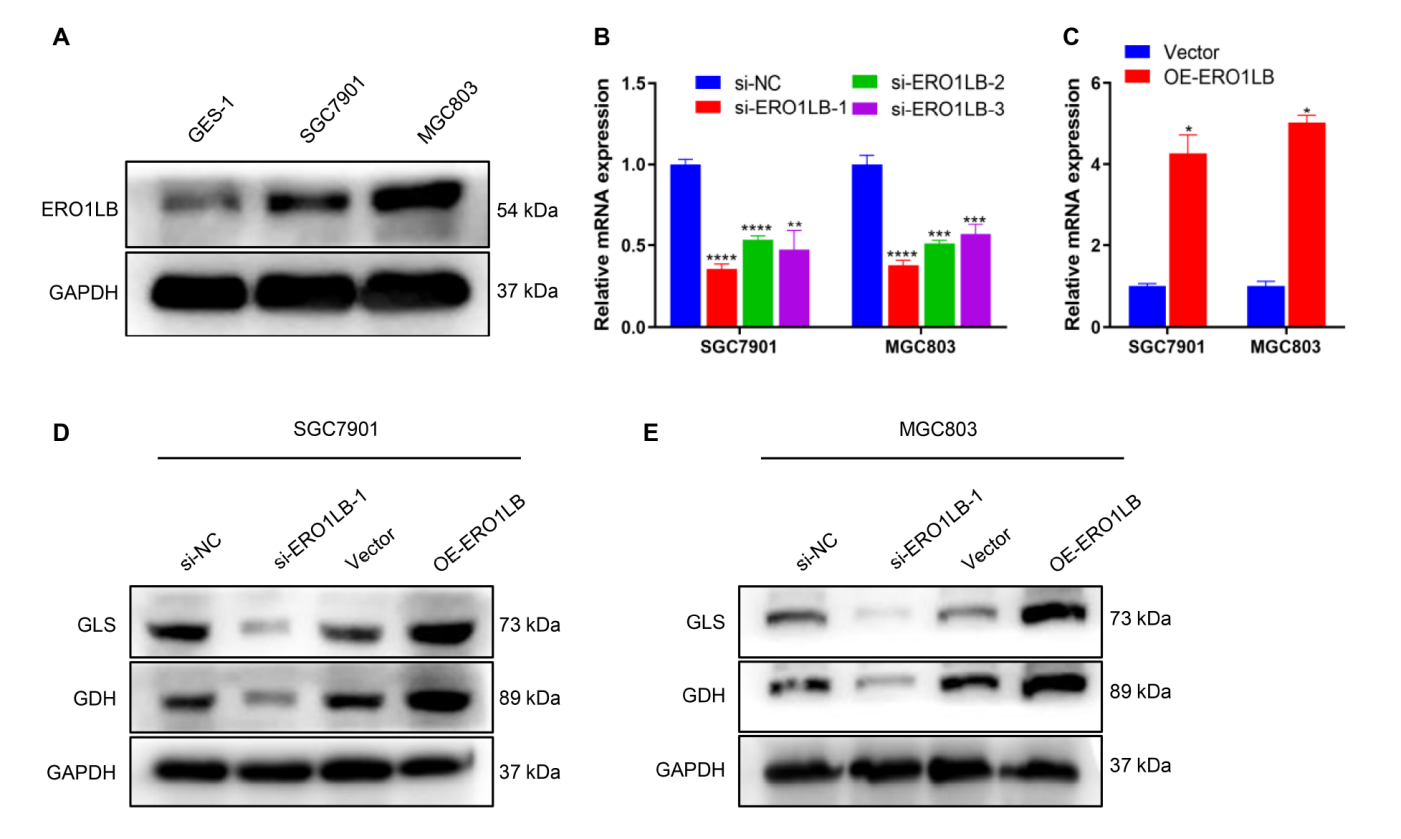
Figure 7 ERO1LB overexpression increases glutamate metabolism in GC cells (A) Representative western blots of ERO1LB expression in GES-1, SGC7901 and MGC803 cells. (B) Detection of ERO1LB mRNA expression in SGC7901 and MGC803 cells transfected with si-NC or si-ERO1LB#1-3 via RT-qPCR. (C) Detection of ERO1LB mRNA expression in two GC cell lines transfected with ERO1LB overexpression (OE-ERO1LB) plasmids via RT-qPCR. (D,E) Representative western blots of GLS and GDH expressions in two GC cell lines transfected with si-ERO1LB#1 or OE-ERO1LB plasmids. *P < 0.05, **P < 0.01, ***P < 0.001, ****P < 0.0001.

Higher expression of ERO1LB was also observed in two GC cell lines, SGC7901 and MGC803, than in the gastric mucosa epithelial cell line GES-1 ( Figure 7A), further revealing the overexpression of ERO1LB in GC. To investigate the biological significance of ERO1LB in gastric tumorigenesis, SGC7901 and MGC803 cells were transfected with ERO1LB siRNAs to downregulated ERO1LB expression ( Figure 7B). In addition, ERO1LB was overexpressed via the transfection of ERO1LB overexpression plasmids into two GC cell lines ( Figure 7C). Glutamine metabolism-related proteins (GLS and GDH) were strongly downregulated in SGC7901 and MGC803 cells in the context of *ERO1LB* knockdown ( Figure 7D,E). In contrast, GLS and GDH expression levels were notably elevated in ERO1LB-overexpressing GC cells. These data revealed that ERO1LB overexpression increased glutamate metabolism in GC cells, thus contributing to tumor progression.

### ERO1LB overexpression reinforces migration and proliferation and attenuates apoptosis in GC cells

**Figure 8:**
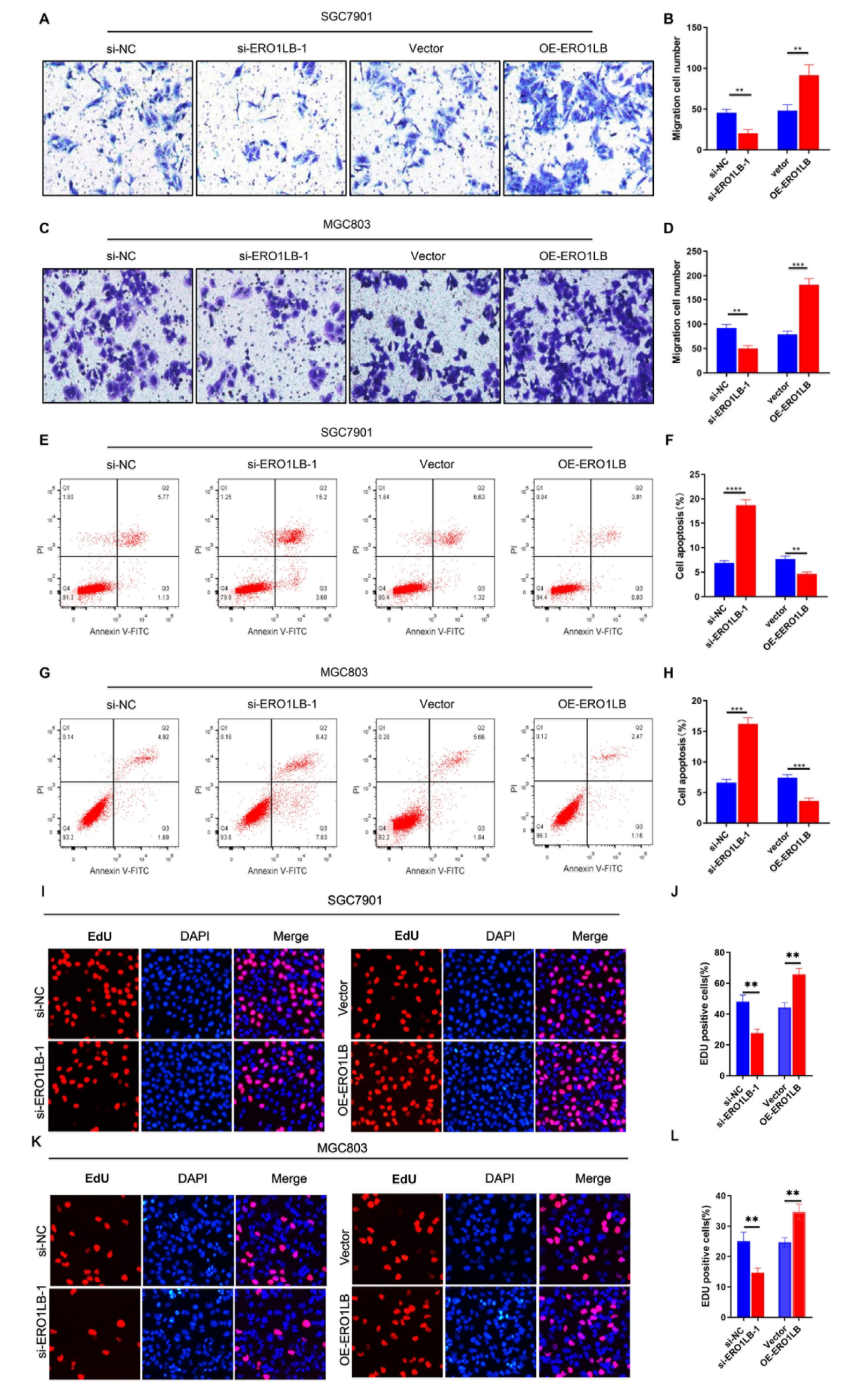
Figure 8 Overexpression of ERO1LB improves migration capacity and alleviates apoptosis in GC cells (A–D) Transwell assay for the migration of SGC7901 and MGC803 GC cells transfected with si-ERO1LB#1 or OE-ERO1LB plasmids. (E–H) Analysis of apoptotic levels in two GC cell lines transfected with si-ERO1LB#1 or OE-ERO1LB plasmids via flow cytometry. (I–L) EdU incorporation assays were performed to examine ERO1LB-mediated cell proliferation. **P < 0.01, ***P < 0.001, ****P < 0.0001.

Subsequent Transwell assays demonstrated that the knockdown of *ERO1LB* impaired the migration capacity of SGC7901 and MGC803 GC cells, whereas its overexpression contributed to the increased migration capacity of GC cells ( Figure 8A–D). In addition, the level of apoptosis was elevated in *ERO1LB*-silenced GC cells and attenuated in ERO1LB-overexpressing GC cells ( Figure 8E–H). Additionally, more EDU-positive ERO1LB-overexpressing cells than *ERO1LB*-silenced cells were detected via EdU assay **(** Figure 8I–L). The above data suggest the involvement of ERO1LB in GC pathogenesis.

### ERO1LB facilitates GC growth and metastasis *in vivo*

**Figure 9:**
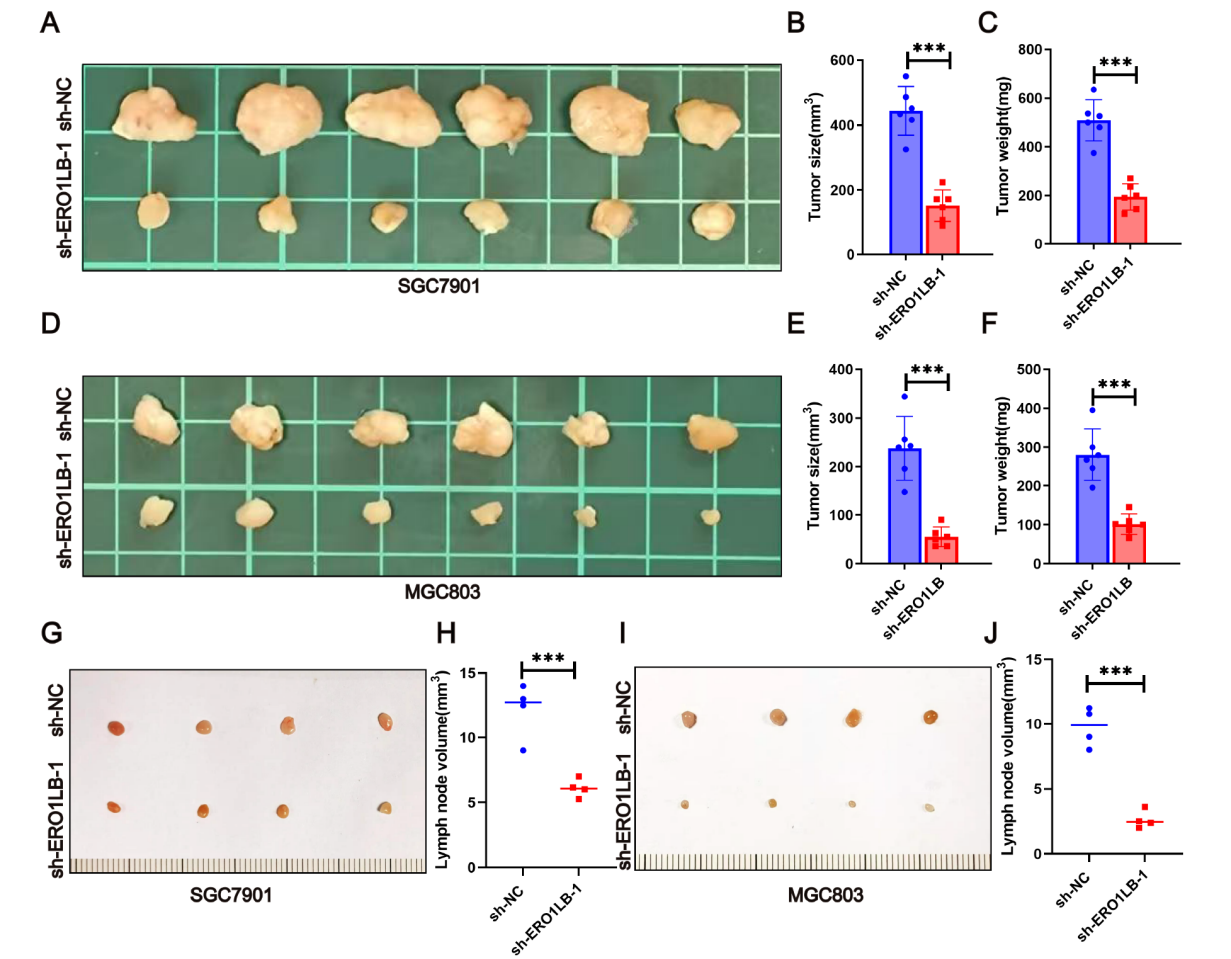
Figure 9 ERO1LB enhances GC growth and lymphatic metastasis *in vivo* (A–F) Representative images showing tumors from nude mice subcutaneously injected with stable ERO1LB-silenced GC cells and quantification of tumor size and tumor weight (n = 6). (G,I) Representative images of popliteal LNs dissected from mice. (H,J) Quantification of the volume of LNs in each group. ***P < 0.001.

To further examine the role of ERO1LB in GC proliferation *in vivo*, GC cells stably transfected with sh-ERO1LB or the corresponding control sh-NC were inoculated subcutaneously into nude mice. The mice were sacrificed five weeks after inoculation. We observed that the size and weight of tumors from nude mice inoculated with *ERO1LB*-knockdown SGC7901 cells were less evident than those from control SGC7901 cells ( Figure 9A–C). Similarly, xenografts derived from MGC803 cells with stable *ERO1LB* knockdown were smaller and lighter than those derived from control MGC803 cells ( Figure 9D–F). To detect the metastatic characteristics of ERO1LB *in vivo*, we constructed a lymph node (LN) metastasis model in nude mice. The sizes of the LNs revealed that *ERO1LB* knockdown abolished LN metastasis ( Figure 9G–J). Collectively, these results suggested that ERO1LB promoted GC proliferation and metastasis *in vivo*.

## Discussion

CAG promotes EGC development in association with chronic inflammation [37]. Cell features are the keys to identifying gastric premalignant and malignant lesions. Nevertheless, how cell type alterations occur during gastric tumorigenesis and how altered cell types lead to the progression of gastric preneoplasia are still unclear. In the healthy gastric mucosa, the epithelium is made up of different cell lineages, comprising mucous cells, secretory cells, and endocrine cells, which work together to sustain tissue homeostasis [19]. The damage to the gastric mucosa induced by *Helicobacter pylori* infection and other risk factors manifests as partial loss of glandular cells, total loss, and the development of CAG. With the progression of CAG, intestinal metaplasia occurs in the gastric mucosa, and several metaplastic cell lineages appear. Hence, comprehensively characterizing cell populations in the gastric mucosa of gastric premalignant and malignant lesions is important. In the present study, a single-cell transcriptome map of CAG and EGC lesions was established, covering epithelial cells, fibroblasts, endothelial cells, T cells, B cells, NK cells and macrophages. Our study revealed the cellular heterogeneity between CAG and EGC lesions. Among these cell types, epithelial cells were found to be the dominant cell type in the two disease states. Notably, the number of epithelial cells was greater in EGCs than in CAGs, indicating the possible dynamic change in epithelial cells during gastric tumorigenesis from CAGs.

Glutamine is a carbon source for lipid and metabolite synthesis through the TCA cycle and is a nitrogen source for the synthesis of amino acids and nucleotides [38]. As a hallmark of cancer metabolism, enhanced glutamate metabolism has been reported to be associated with GC progression [8], metastasis [39], therapeutic therapy [9], *etc*. Increased activity of glutamate metabolism is also observed in CAG [ 14, 40] . Although glutamine metabolism mediates CAG-induced carcinogenesis, the molecular mechanisms governing this process are unclear [14]. The present study revealed dynamic alterations in glutamine metabolism in premalignant and early-malignant gastric lesions. Notably, glutamine metabolism activity was greater in epithelial cells in EGCs than in those in CAG, and among the epithelial cell subpopulations, glutamine metabolism activity was greater in the epithelial cell subpopulation cluster_4 in EGCs than in those in CAG lesions, revealing the potential contribution of glutamine metabolism in epithelial cells to CAG-induced carcinogenesis. Nonetheless, the underlying molecular mechanisms need in-depth exploration.

This study identified CHGA, CHGB, CPE, ERO1LB, MDK, MS4A8, PAM, PCSK1N, SCG5, TM4SF4 and TUBA1A as novel markers of epithelial cell subpopulation cluster_4. Among them, we focused on ERO1LB, which enables thiol oxidase activity, reinforces insulin biogenesis and glucose homeostasis, and mediates protein folding in the endoplasmic reticulum [ 34– 36] . Previous studies have demonstrated the involvement of ERO1LB in several human cancer types. For example, *ERO1LB* is a key gene associated with tumor-infiltrating plasma cells in lung adenocarcinoma [41]. In addition, ERO1LB is linked to breast cancer metastasis [42]. This study revealed a novel ERO1LB ^+^ epithelial cell subpopulation that might be responsible for the malignant transformation from CAG to EGC. Targeted suppression of ERO1LB in GC cells suppressed cell growth and migration, triggered apoptosis and weakened glutamate metabolism, and vice versa. Our study revealed the potential of ERO1LB as a therapeutic target in GC.

Here we investigated the cellular heterogeneity of glutamate metabolism in premalignant and malignant gastric lesions. Despite these findings, our study has several limitations: the carcinogenic mechanism of ERO1LB in CAG is an intricate process, and our study provided only preliminary evidence. More studies are needed to verify the role of ERO1LB in CAG-induced carcinogenesis. Moreover, we should further investigate the underlying mechanisms of ERO1LB in GC progression.

In summary, in this study we established a single-cell transcriptome landscape of gastric premalignant and early-malignant lesions. In the landscape, we dissected the glutamate metabolism characteristics of distinct cell populations in the two gastric lesions. Notably, *ERO1LB* acts as a key marker gene of the epithelial cell subpopulation cluster_4, and its overexpression enhances GC cell glutamate metabolism; facilitates growth and migration; and prevents apoptosis. Our findings may contribute to the development of early intervention regimens for GC and deepen our understanding of GC pathogenesis.

## Supporting information

24916Supplementary_Figures

## Supplementary Data

Supplementary Data is available online at *Acta Biochimica et Biophysica Sinica*.
