# Supplementary material for: Single-cell transcriptomic data reveal the cellular heterogeneity of glutamine metabolism in gastric premalignant lesions and early gastric cancer: Single-cell transcriptomic data reveal the cellular heterogeneity of glutamine metabolism
Source: Acta Biochim Biophys Sin (Shanghai). 2025 Apr 23;57(10):1670–83. doi: 10.3724/abbs.2025061 (PMC12616717; doi:10.3724/abbs.2025061)
Supplement: 24916Supplementary_Figures [file 24916Supplementary_Figures.docx]

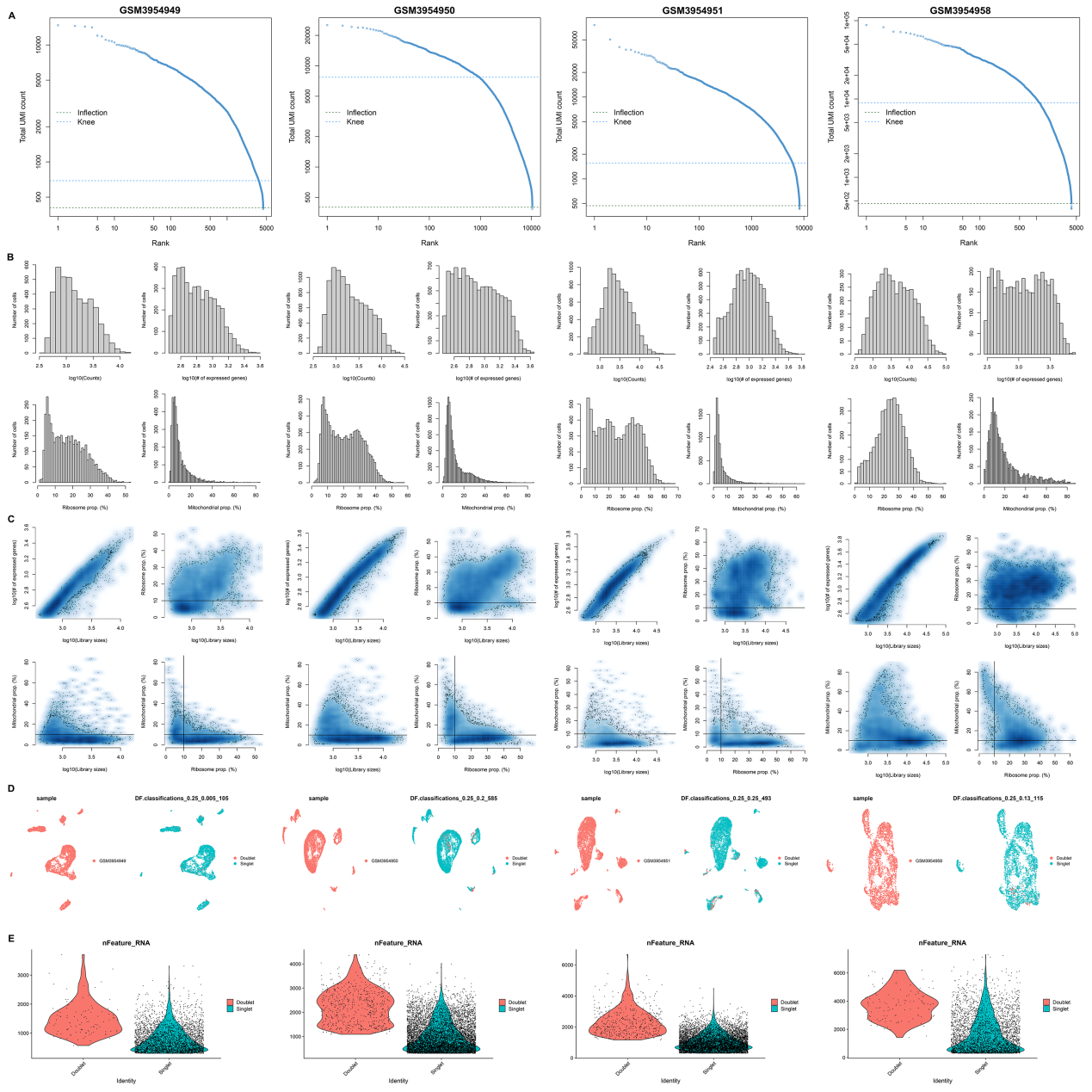


**Supplementary Figure S1. Quality control and preprocessing on single-cell transcriptomics from three CAG and one EGC specimens**  (A) Barcode rank plots depicting the inflection points of curves. The first inflection point shows a rapid transition from high to low UMI. Higher UMI indicates a real cell. (B) Number of cells that expressed diverse proportions of mitochondrial or ribosome genes. (C) Distribution of proportions of mitochondrial or ribosome genes. (D,E) Detection and removal of doublets.


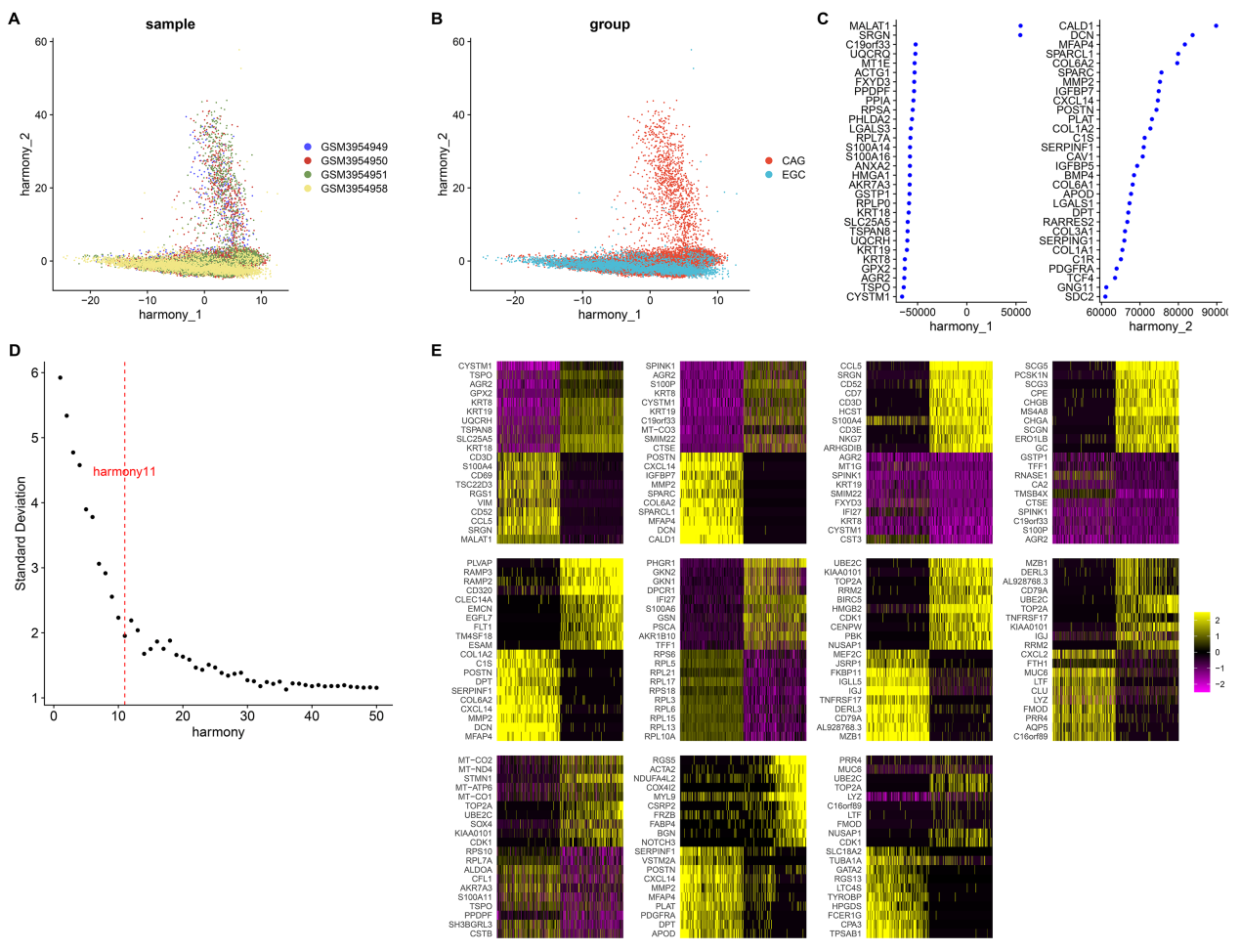


**Supplementary Figure S2. Dimension reduction analysis on single-cell transcriptomics** (A,B) Visualization of single cells from three CAG and one EGC lesions. (C) The major contributors of the first two principal components. (D) Selection of 11 as the appropriate number of principal components. (E) The major contributors of the first 11 principal components.

**
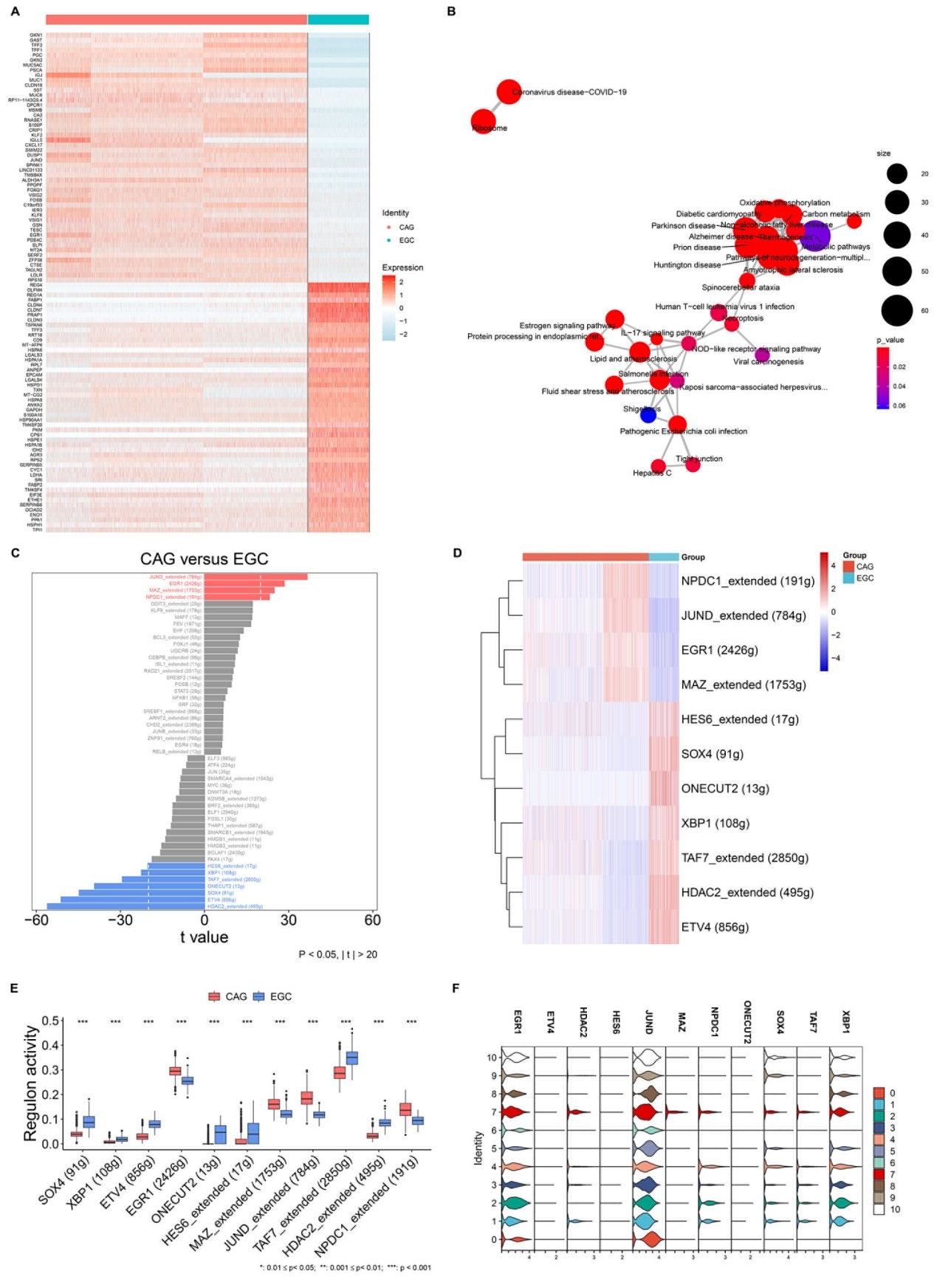
**

**Supplementary Figure S3. Transcriptional regulatory programs in epithelial cells of premalignant and early-malignant lesions** (A) Heatmap illustrating differential expression in epithelial cells of CAG and EGC. (B) KEGG pathway network based upon the genes with differential expression. (C) Differential transcriptional factors in epithelial cells between CAG and EGC. (D) Differential expression patterns of transcriptional factors in CAG versus EGC epithelial cells. (E) Comparison of regulon activity in epithelial cells between CAG and EGC. (F) Distribution of regulon activity across diverse epithelial cell subpopulations. ****P* < 0.001.


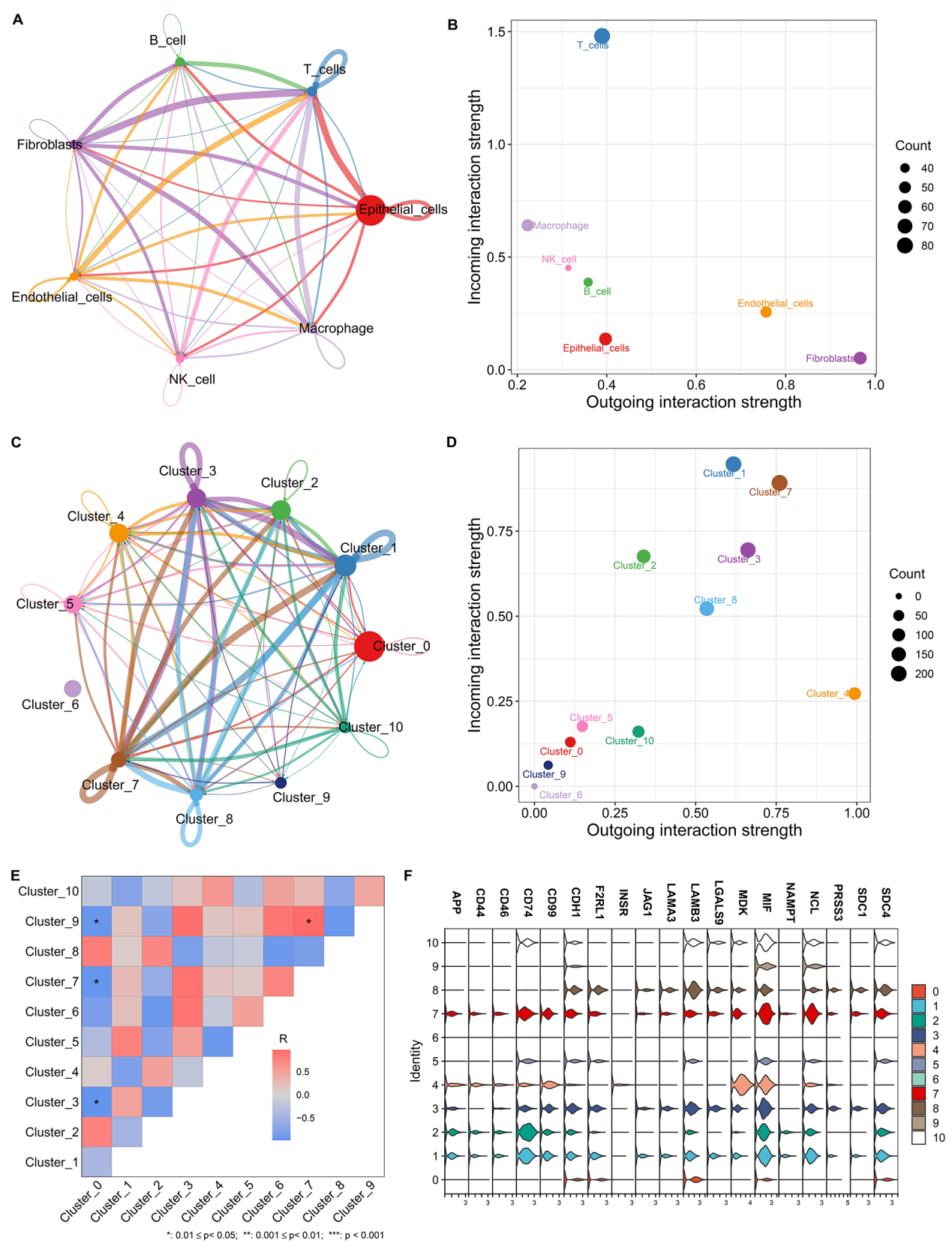


**Supplementary Figure S4. Intercellular communications in the inflammatory microenvironment**  (A) Network of communications between diverse cell types. (B) Incoming and outcoming interaction strength of each cell type. (C) Network of communications between epithelial cell subpopulations. (D) Incoming and outcoming interaction strength of each epithelial cell subpopulation. (E) Correlation analysis on different epithelial cell subpopulations. (F) Expression of ligands and receptors across epithelial cell subpopulations. **P* < 0.05.
